# Approaches, tools and methods used for setting priorities in health research in the 21^st^ century

**DOI:** 10.7189/jogh.06.010507

**Published:** 2016-06

**Authors:** Sachiyo Yoshida

**Affiliations:** Department of Maternal, Newborn, Child and Adolescent Health, World Health Organization, Geneva, Switzerland

## Abstract

**Background:**

Health research is difficult to prioritize, because the number of possible competing ideas for research is large, the outcome of research is inherently uncertain, and the impact of research is difficult to predict and measure. A systematic and transparent process to assist policy makers and research funding agencies in making investment decisions is a permanent need.

**Methods:**

To obtain a better understanding of the landscape of approaches, tools and methods used to prioritize health research, I conducted a methodical review using the PubMed database for the period 2001–2014.

**Results:**

A total of 165 relevant studies were identified, in which health research prioritization was conducted. They most frequently used the CHNRI method (26%), followed by the Delphi method (24%), James Lind Alliance method (8%), the Combined Approach Matrix (CAM) method (2%) and the Essential National Health Research method (<1%). About 3% of studies reported no clear process and provided very little information on how priorities were set. A further 19% used a combination of expert panel interview and focus group discussion (“consultation process”) but provided few details, while a further 2% used approaches that were clearly described, but not established as a replicable method. Online surveys that were not accompanied by face–to–face meetings were used in 8% of studies, while 9% used a combination of literature review and questionnaire to scrutinise the research options for prioritization among the participating experts.

**Conclusion:**

The number of priority setting exercises in health research published in PubMed–indexed journals is increasing, especially since 2010. These exercises are being conducted at a variety of levels, ranging from the global level to the level of an individual hospital. With the development of new tools and methods which have a well–defined structure – such as the CHNRI method, James Lind Alliance Method and Combined Approach Matrix – it is likely that the Delphi method and non–replicable consultation processes will gradually be replaced by these emerging tools, which offer more transparency and replicability. It is too early to say whether any single method can address the needs of most exercises conducted at different levels, or if better results may perhaps be achieved through combination of components of several methods.

Apart from the continuing need to prioritize investments in health systems and health interventions, there is also a need to prioritize health research. Health research is difficult to prioritize, because the number of possible competing ideas for research is large, the outcome of research is inherently uncertain, and the impact of research is difficult to predict and measure [1]. A systematic and transparent process to assist policy makers and research funding agencies in making investment decisions is a permanent need.

At national level several methods have been tried: some of the best examples are the Council on Health Research for Development’s approach (COHRED) in Brazil, Cameroon, Peru and Philippines; the Essential National Health Research (ENHR) approach in Cameroon and South Africa; and the Combined Approach Matrix (CAM) in Malaysia, Pakistan and Argentina [2,3]. COHRED, ENHR and CAM were all developed by committees set up by international agencies, such as the World Health Organization (WHO) or the Global Forum for Health Research (GFHR). These methods are useful for organizing the available information so that the research prioritization can take place.

To obtain a better understanding of the landscape of approaches, tools and methods used to prioritize health research I conducted a methodical review of the PubMed database covering the period 2001–2014. My primary aim was not to perform an exhaustive review of the field, which would include searching all available scientific databases and grey literature. Instead, I was interested in identifying the methods and tools that are being commonly used in the papers that are most readily accessible through databases in the public domain such as PubMed, and to assess their relative importance and applicability. The review of PubMed for the period between 2001 and 2014 achieves this aim, because this limits the search of priority–setting tools to health topics only, which is the main interest of this analysis, while drawing on a very large database which is publically available and which should contain the vast majority of relevant studies.

## METHODS

**Figure 1:**
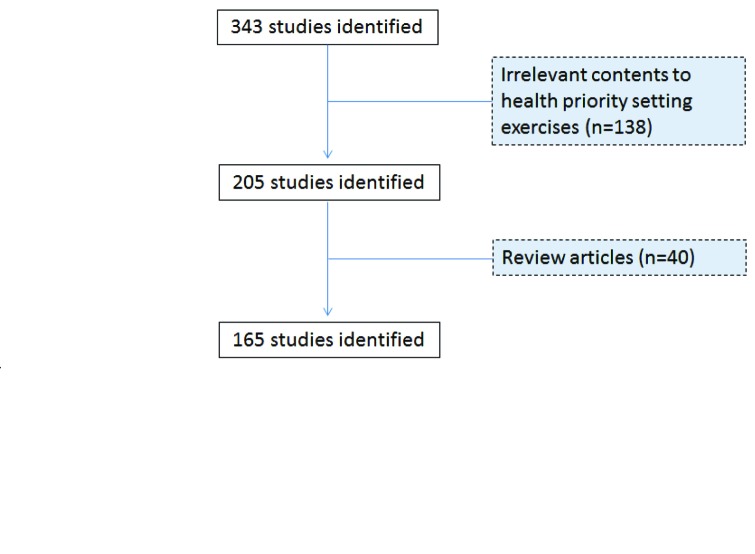
Flowchart of the review on all priority–setting exercises for health research conducted between 2001 to 2014.

My search terms included “research priorit* OR priorit* research”. These terms were chosen as the most informative combination of search terms after experimenting with several versions of search terms. The search terms identified 343 publications, 138 of which were excluded from the analysis because their contents were irrelevant to health research priority setting. A further 40 studies were excluded because they were review articles which did not attempt to set priorities. In total, 165 relevant studies were identified and retained for the analysis. **Figure 1** shows a flowchart of the review on all research priority setting exercises conducted between 2001 and 2014.

## RESULTS

**Figure 2:**
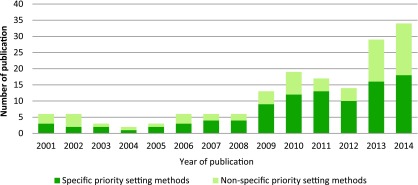
Total number of publication by year (source: PubMed, 2001 to 2014).

**Figure 3:**
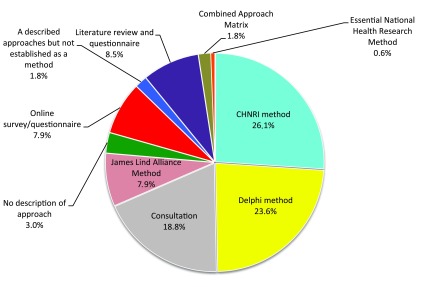
Methods, tools and approaches used for setting health research priorities (source: PubMed, 2001 to 2014).

Approximately 12 exercises were initiated each year between 2001 and the end of 2014. Since 2012, there has been a steady increase in the number of exercises published with the peak in 2014 with 34 exercises published (**Figure 2**). Of the 165 publications identified, the most frequently used was the CHNRI method (26%), followed by the Delphi method (24%), James Lind Alliance method (8%), the Combined Approach Matrix (CAM) method (2%) and the Essential National Health Research method (<1%). COHRED method, although frequently mentioned and clearly described in the historic context of national–level research priority setting, was not underlying any specific priority–setting process in the time period which I studied. Online surveys that were not accompanied by face–to–face meetings were used in 8% of studies, while 9% used a combination of literature review and questionnaire to scrutinise the research options for prioritization among the participating experts. About 3% of studies reported no clear process and provided very little information on how priorities were set. A further 19% used a combination of expert panel interview and focus group discussion (“consultation process”) but provided few details, while a further 2% used approaches that were clearly described, but not established as a replicable method (**Figure 3**). At this point, I would like to clarify that “replicable” refers to the method's description in sufficient detail, so that all other users could apply it in the same way. It does not refer to method's property to yield the same results when repeated, which is a different meaning of the term “replicable” when assigned to a method.

**Tables 1** to **6** provide a brief description of the approaches and processes used by the specific methods mentioned in **Figure 3**. The methods range from those that are not described at all, through vaguely described processes of group decision making, to those that follow a certain structure/process and use transparent criteria. Their output is typically quite general, ie, pointing to broad research areas in which more research activity is needed. As described above, COHRED, ENHR and CAM are used in assembling the evidence that can be used for the consultation but not for the ranking of priorities. Nevertheless, the use of any method, regardless of its limitations, is preferable to the alternative of having no clearly defined approach at all [3].

**Table 1:** Brief explanation of the Essential National Health Research (ENHR) [4–6]

Overall process	ENHR was developed by Commission on Health Research for Development in 1990. It is a step by step guide for national research priority setting, focused on equity in health and development. Strategy focused on inclusiveness in participation, broad–based consultations at different levels, both quantitative and qualitative information used, and stewardship by small working group.
How are participants identified?	Participants are involved through a small representative working group which can facilitate the process, through various consultations. These stakeholders have a major stake in the goal of equity in health and development. The four major categories of participants include: researchers, decision makers, health service providers and communities.
How are research ideas identified	Stakeholders suggest priority areas, via evidence based situation analysis (such as looking at health status, health care system, health research system). Research ideas are gathered from a nomination process from different stakeholders. Consensus building using methods such as brainstorming, multi–voting, nominal group technique, round–table is then used to select research ideas.
Scoring criteria	Criteria is selected as to be: – Appropriate to the level of the action of the priority setting *i.e.* global, national, district; – Detailed in definition; – Independent of each other; – Contain information base; – Reflect equity promotion and development; – Manageable number; – Expressed in a common language. Criteria are agreed on by brainstorming of large collection of possible criteria, eliminating duplicates and clearly defining the meaning of each criterion from stakeholders. Criteria will then be put into representative categories and finally selected depending on purpose and level of action of priority–setting exercise.
Scoring options	Each criteria is scored: Point score to each criteria OR Number of score choices to each criteria
Advantages	– Broad based inclusion and participation of different stakeholders. – Multidisciplinary and cross–sectoral approach – Partnership development – Transparent process – Systematic analyses of health needs
Disadvantages	– Vague criteria and lack of transparency in individual process used by countries – Few countries had guidelines on how to develop nor apply criteria – Needs stronger representation of groups such as private sector, parliamentarians, donors, international agencies– Does not provide methodology for identifying participants

**Table 2:** Brief explanation of the Combined Approach Matrix (CAM) [7,8]

Overall process	Developed by the Global Forum for Health Research, CAM was to bring together economic and institutional dimensions into an analytical tool with the actors and factors that play a key role in health status of a population. It also aims to organise and present a large body of information that enters the priority setting process. This will help decision makers make rational choices in investment to produce greatest reduction in burden of disease.
How are participants identified?	Institutional approach involving: individual, household and community; health ministry and other health institutions; other sectors apart from health; and macroeconomic level actors.
How are research ideas identified	Five step process including measuring the disease burden, analysing determinants, getting present level of knowledge, evaluating cost and effectiveness, and present resource flows. For each main disease and risk factor, institutions and stakeholders with particular knowledge are brought together to provide information via workshops and brainstorming. Each institution will feed into matrix the information at disposal, regarding a specific disease or factor; the matrix will reveal how little information is available in some areas which can then be candidates for research. Each participant determined the priority research topics based on CAM evidence, then grouping the topics and cutting down to establish the top priorities.
Scoring criteria	Criteria based on questions of what is a research priority in the context, and what is not known but should be.
Scoring options	N/A
Advantages	– Creates framework of information – Identifies gaps in knowledge – Facilitates comparisons between sectors – Broad inclusion of actors – 3D–CAM includes equity
Disadvantages	– Difficult and time–consuming as involves multi–stage discussion – Does not provide algorithm to establish and score research priorities therefore is not repeatable nor systematic – Does not provide methodology for identifying participants

**Table 3:** Brief explanation of the James Lind Alliance Method [9]

Overall process	Focuses on bringing patients, carers and health professionals in order to identify treatment uncertainties which will become research questions. The method uses a mixture of data gathering, quantitative and qualitative analysis to create research priorities in areas of treatment uncertainty.
How are participants identified?	Participants are identified through Priority Setting Partnerships which brings patients, carers and clinicians equally together and agree through consensus priorities.
How are research ideas identified	Treatment uncertainties are defined as no up to date, reliable systematic reviews addressing treatment uncertainty, or systematic review that shows such uncertainty exists. Step 1: Recommendations by PSPs, or through looking at existing literature, creates a list of uncertainties. Step 2: These are then verified through systematic reviews of databases to verify they are research gaps using Cochrane, DARE, NICE, Sign. An uncertainty is deemed genuine when a reported confidence interval in a systematic review does not cross the line of effect or line of unity. A virtual interim priority ranking, and a final priority setting workshop takes place to agree upon 10 prioritised uncertainties through consensus building.
Scoring criteria	No clear criteria are identified with which to use.
Scoring options	Ranked AND Qualitative consensus
Advantages	– Takes into account underrepresented groups – Applicable to small scale prioritisation (eg, hospital) – Mixture of methods
Disadvantages	– Time consuming to identify and verify treatment uncertainties – Selection of criteria not clear – Not suitable for global level, nor specific disease domains – Very clinically orientated – Disproportionate mix of participants may skew information base

**Table 4:** Brief explanation of the Council on Health Research for Development (COHRED) [10]

Overall process	COHRED uses a management process for national level exercises to show important steps for priority setting processes.
How are participants identified?	Participants are identified through the chosen methods outlined in the steps of the COHRED guide.
How are research ideas identified	Identification of priority issues much choose method best suited to local context and needs either through compound approaches (ENHR, CAM, Burden of Disease) or foresighting techniques (Visioning, Delphi). Consider using more than one method to optimize usefulness of results.
Scoring criteria	COHRED presents ranking techniques that can be used to rank priority issues including direct and indirect valuation techniques.
Scoring options	Ranked
Advantages	– Overview approach providing steps – Discusses wide range of options – Flexible to contexts and needs
Disadvantages	– Too general and unspecific – Lack of criteria transparency

**Table 5:** Brief explanation of the Delphi Process [11]

Overall process	Delphi, mainly developed in the 1950s, is a systematic, interactive forecasting method which relies on a panel of experts and questionnaires.
How are participants identified?	Participants are eligible to be invited if they have related backgrounds and experiences concerning the target issue, are capable of contributing, and are willing to revise their initial judgements in order to reach consensus. Participants are considered and selected through investigators, ideally through a nomination process, or selection from potential leaders or authors through publication. It is suggested that the three groups are used: top management decision makers who will utilise outcomes of Delphi study; professional staff members and their support team; respondents to the Delphi questionnaire. It is recommended to use the minimally sufficient number to generate representative pooling of judgements – however no consensus yet as to optimal number of subjects.
How are research ideas identified	In the first round an open–ended questionnaire is sent to solicit information about a content area from Delphi participants. Investigators will then turn the responses into a well–structured questionnaire to be used as survey for data collection. Through four rounds experts answer questionnaires; the facilitator summarises anonymously the forecast after the first round and the experts are then asked to revise their earlier answer thereby decreasing the range of answers and converging towards the correct answer. Up to four iterations can be used.
Scoring criteria	N/A
Scoring options	Rate or ranking AND Consensus building
Advantages	– Multiple iterations and feedback process – Flexible to change – Anonymity of respondents
Disadvantages	– Does not provide methodology for identifying participants – Lack of criteria transparency – Potential for low response rate due to multiple iterations – Time–consuming – Potential for investigators and facilitators to bias opinions

**Table 6:** Brief explanation of the CHNRI process [12–15]

CHNRI method Child Health Nutrition Research Initiative	
Overall process	The CHNRI methodology was introduced in 2007 by the Child Health and Nutrition Research Initiative of the Global Forum for Health research. The methodology was developed to address gaps in the existing research priority methods. The CHNRI method is developed to assist decision making and consensus development. The method include soliciting ideas from different carder of participants on the given health topic and use independent ranking system against the pre–defined criteria to prioritise the research ideas.
How are participants identified?	Participants are identified by management team based on their expertise (eg, number of publications, experience in implementation research and programmes etc). Participants includes stakeholders who might not have the technical expertise but have view on the health topic of concern.
How are research ideas identified?	Research ideas are generated by participants or by management team based on the current evidence. If former, usually each participant is asked to provide maximum of three research questions against the predefined domain of health research (eg, descriptive research, development research, discovery research and delivery research). The ideas are usually submitted via online survey and consolidated by the management team.
Scoring criteria	Five standard criteria are usually used: – Answerability – Equity – Impact on burden – Deliverability – Effectiveness. Though standard criteria is used more than half of the exercises it is flexible to add or remove criteria depending on the needs of the exercise.
Scoring options	Each criteria is scored: Point score to each criteria in the scale of 0, 0.5 and 1 or in the scale of 0 to 100.
Advantages	– Simple, inclusive and replicable and thus systematic and transparent process. – Independent ranking of experts (avoid having the situation where one strongly minded individual affecting the group decision) – Less costly
	– Potentially represent collective opinion of the limited group of people who were included in the process. – Scoring affected by currently on–going research

Among the 165 identified studies that set health research priorities, 21% were conducted at global level, 50% of the exercises were focused on High Income Countries (HICs) and 28% were focused on Low and Middle Income Countries (LMIC). At the national level, the countries where research priority exercises were most frequently initiated were the UK (27%), USA (16%), Australia (15%), and Canada (11%) (**Table 7**).

**Table 7:** Distribution of identified studies by geographic context and countries where the research priority setting exercises have been initiated and research priority areas addressed

Geographical area	Number	%	Technical areas	Number	%
Global	35	21	Non–communicable disease	29	18
High income countries	82	50	Child and adolescent health	28	17
Low middle income countries	47	28	Mental health	16	10
Humanitarian settings	1	<1	Infectious disease	14	8
**TOTAL**	**165**	**100**	Nursing/Midwifery	13	8
**National level**			Public health in general	10	6
Australia	15	15	Policy and health system	8	5
Brazil	1	1	Occupational health/therapy	6	4
Canada	11	11	Reproductive health/women's health	6	4
Colombia	1	1	Skin disease	5	3
Chile	1	1	Emergency care	3	2
Cuba	1	1	Environmental health	3	2
Hong Kong	2	2	Disability	3	2
India	1	1	Child development potential	2	1
Iran	2	2	Injury prevention	2	1
Ireland	3	3	Maternal and perinatal health	2	1
Italy	1	1	Pharmaceuticals	2	1
Malaysia	1	1	Microbial Forensics	2	1
Nepal	1	1	Behavioural science	1	1
The Netherlands	1	1	Diagnostic accuracy	1	1
Nigeria	1	1	Tuberculosis	1	1
Peru	1	1	Medical science	1	1
Portugal	2	2	Neurological	1	1
South Africa	3	3	Nutrition	1	1
Saudi Arabia	1	1	Surgical	1	1
Spain	3	3	Surveillance system	1	1
United Republic of Tanzania	2	2	Water and sanitation	1	1
United Kingdom	26	27	Primary health care–related disease	1	1
United States of America	16	16	Others	1	1
**TOTAL**	**97**	**100**	**TOTAL**	**165**	**100**

Topic areas for which research priorities were identified included non–communicable diseases (18%), followed by child and adolescent health (17%), mental health (10%), nursing/midwifery (8%) and infectious disease (8%). The remaining exercises (39%) covered a wide variety of topics, including policy and health system, occupational health /therapy, reproductive health/women’s health, emergency care, environmental health, occupational health, forensic science and injury prevention (**Table 7**).

## DISCUSSION

The number of priority setting exercises in health research published in PubMed–indexed journals is increasing, especially since 2010. These exercises are being conducted at a variety of different levels, ranging from the global level to the level of an individual hospital. With the development of new tools and methods which have a well–defined structure – such as the CHNRI method, James Lind Alliance Method and Combined Approach Matrix – it is likely that the Delphi method and non–replicable consultation processes (see the definition of “replicable” earlier in the text) will gradually be replaced by these emerging tools, which offer more transparency and replicability. This is a process that should be endorsed, as a natural progression of the priority–setting field from the period in which hardly any structured processes existed to fill a need, to the new era which will be increasingly dominated by structured and well–defined tools.

This review is not the first attempt to assess approaches, tools and methods to set health research priorities. Searching the literature, I identified five earlier attempts to review and discuss priority–setting processes. The first review was published by Rudan and colleagues in 2007 in an attempt to develop an evidence base for the development of conceptual framework and guidelines for implementation of the CHNRI methodology [1]. This paper identified ambitious attempts by several large organizations at the international level to define health research priorities for either the whole developing world, large world regions or nationally. These attempts date back to the year 1990, with the “...Commission on Health Research for Development usually being referred to as the first truly significant international initiative aimed toward systematic approach to setting priorities in global health research.” Other initiatives that followed were the “Ad Hoc Committee (AHC) on Health Research Relating to Future Intervention Options” (in 1994), the “Global Forum for Health Research” (in 1998), the “Council on Health Research and Development (COHRED)” (in 2000), “The Grand Challenges” proposed at the World Economic Forum in Davos, Switzerland (in 2003) and the “Combined Approach Matrix” as the first specific priority–setting tool for health research (in 2004). The paper concluded that the processes, initiatives and tools fell short of being informative on what the specific research priorities should be and how exactly are they derived [1].

In 2010, Viergever et al. [16] reviewed the articles that set health research priorities and they specifically reviewed exercises coordinated by World Health Organization Headquarters since 2005. This resulted in the total of 230 documents or reports, many of them unpublished (hence, not included in my review). The authors concluded that, at that point in time, there was no “gold standard” approach for health research prioritisation. This was not surprising, given the heterogeneity in the context of research prioritization exercises and different levels at which they were being conducted. Nevertheless, the authors observed several common themes of “good practice” and proposed a generic framework – in the form of “checklist”, like a form of “guidelines” – which also suggested various options for each step of the process. Nine themes were identified through a review of the previously conducted priority–setting processes. They were categorized as the “themes during the preparatory work” (defining context, use of comprehensive approach, ensure inclusiveness of participants, information gathering, planning for implementation), followed by the steps in the process of deciding on the priorities (defining the criteria, methods for deciding on priorities), and two steps in the last phase after the priorities have been set (plan the timing of evaluation in terms of how the research priorities are being used, and write the clear report of the methodology used to ensure the transparency in the process). The authors proposed that the provision of the framework should be of assistance to policy makers and researchers. It could have a dual role: it could not only assist priority–setting process, but also planning the follow up and implementation of the priorities [16].

In the same year, in 2010, the World Health Organization's Department for Research Policy and Cooperation held a consultation between methodology–developing experts to identify optimal characteristics of priority–setting methods that could be applicable at the national level. The aim was to empower low and middle–income countries to take more ownership of their own health research agenda. Tomlinson reviewed the progress made at this meeting and published the main conclusions in 2011 [2]. Three methods emerged as applicable at the national level: the Combined Approach Matrix (CAM), the Council on Health Research and Development (COHRED) and the Child Health and Nutrition Research Initiative (CHNRI). The authors presented and discussed strengths and weaknesses of each method [2]. They also noted that, across the countries surveyed, genuine engagement of stakeholders was difficult to achieve and was typically missing. Countries also varied in the extent to which they would document priority–setting processes, with not a single country having an appeal process for outlined priorities. Another problem was that the identified priorities usually outlined broad disease categories, rather than more specific research questions [2]. The authors concluded that priority–setting processes should aim to include mechanisms for publicizing results, effective procedures to translate and implement decisions and processes to ensure that the revision of priorities eventually does occur.

In a more recent report, an independent team from the Kirby Institute in Sydney, Australia, systematically reviewed all studies undertaken in low– and middle–income country (LMIC) settings that attempted to set research priorities over the period from 1966 to 2014. The studies included were not reported but they found 91 studies, including 16 which used the CHNRI method [17]. The authors concluded that almost half of these processes took place at the global level (46%). For regional or national initiatives, a half focused on Sub Saharan Africa (49%), followed by East Asia and the Pacific (20%) and Latin America and the Caribbean (18%). Most commonly, studies were initiated by an international organization or collaboration (46%). Researchers and governments were the most commonly represented stakeholders. The most frequently used process was a conference or workshop to determine priorities (24%), followed by the CHNRI method (18%) [17]. The review revealed inconsistent use of existing methods and approaches in health research prioritization processes. It also showed that while there was strong involvement of government and researchers, participation of other key stakeholders was limited. The authors argued that many processes, regardless of the method used, lacked an implementation strategy to translate the result of the process into implementation of research projects. Finally, the authors concluded that research prioritization exercises would often remain “one–time exercises”, given the lack of follow up and implementation strategies involving the funders, researchers and government officials.

Finally, in 2014, as a part of the Lancet series on increasing value and reducing waste in health research generally, one paper of the series (by Chalmers et al. [18]) explored how to increase value and reduce waste when research priorities are set. The group of authors argued that many basic research endeavours do not lead to knowledge that is useful to the end user of the research results. By using various examples, the authors reiterate the same argument: if research does not meet the needs of the users of research, evidence will have little impact on public health and clinical practice. The authors argue that many research studies that fall in the area of basic (fundamental) research were duplicative. Although a replication of positive findings is a welcome process, an excessive repetition of conducting similar research can be prevented by either: (i) conducting systematic reviews and also involving the end user of the research as well as clinicians in the process (where they used the example of hospital based research priority setting exercise using the James Lind Alliance method); and (ii) mapping research portfolios of major agencies, that could help to prevent duplication in the nature of supported research. The main message of the article is, therefore, a need for better co–ordination among the researchers and the funders over the research that is being conducted and increased focus on the translational value of the information that is being generated through research [18].

It is evident from my own methodical review, and from the systematic review undertaken by the researchers from the Kirby Institute, that there is a need for a transparent, replicable, systematic and structured approach to research priority setting, because the large majority of the previous exercises were not based on processes meeting all of these criteria. The review by McGregor et al. [17] shows how, although a very recent addition to the set of tools, the CHNRI method is set to become the most widely used approach.

The results of my review broadly confirmed the observations of all previous reviews, with an additional insight into time trend – showing an increase in the number of exercises conducted over time, and gradual replacement of poorly defined processes with those that use particular methods and tools, as shown in **Figure 2**. The next step in the field of health research priority setting should therefore involve monitoring whether any single method may address the need for most exercises conducted at different levels, or if better results may perhaps be achieved through combination of strengths of several methods.
